# LINC00958 as new diagnostic and prognostic biomarker of childhood acute lymphoblastic leukaemia of B cells

**DOI:** 10.3389/fonc.2024.1388154

**Published:** 2024-05-31

**Authors:** Filomena Altieri, Lorena Buono, Mariamichela Lanzilli, Peppino Mirabelli, Alessandra Cianflone, Giuliana Beneduce, Antonia De Matteo, Rosanna Parasole, M. Salvatore, Giovanni Smaldone

**Affiliations:** ^1^IRCCS SYNLAB SDN, Naples, Italy; ^2^Department of Paediatric Haemato-Oncology, Santobono-Pausilipon Children’s Hospital, AORN, Naples, Italy

**Keywords:** B-ALL, biomarkers, diagnosis, prognosis, lncRNA, relapse

## Abstract

**Background:**

Paediatric acute B-cell lymphoblastic leukaemia is the most common cancer of the paediatric age. Although the advancement of scientific and technological knowledge has ensured a huge step forward in the management of this disease, there are 15%–20% cases of recurrence leading to serious complications for the patient and sometimes even death. It is therefore necessary to identify new and increasingly personalised biomarkers capable of predicting the degree of risk of B-ALL in order to allow the correct management of paediatric leukaemia patients.

**Methods:**

Starting from our previously published results, we validate the expression level of LINC00958 in a cohort of 33 B-ALL and 9 T-ALL childhood patients, using *in-silico* public datasets as support. Expression levels of LINC00958 in B-ALL patients stratified by risk (high risk vs. standard/medium risk) and who relapsed 3 years after the first leukaemia diagnosis were also evaluated.

**Results:**

We identified the lncRNA LINC00958 as a biomarker of B-ALL, capable of discriminating B-ALL from T-ALL and healthy subjects. Furthermore, we associated LINC00958 expression levels with the disease risk classification (high risk and standard risk). Finally, we show that LINC00958 can be used as a predictor of relapses in patients who are usually stratified as standard risk and thus not always targeted for marrow transplantation.

**Conclusions:**

Our results open the way to new diagnostic perspectives that can be directly used in clinical practice for a better management of B-ALL paediatric patients.

## Background

Acute lymphoblastic leukaemia of B cell, also known as B-ALL, is a type of leukaemia that arises from abnormal proliferation of precursor of B lymphocytes in the bone marrow (1). B-ALL is the most common cancer of paediatric age, but it can occur in adults as well. The exact cause of childhood ALL is not well understood. However, research suggests that it may result from a combination of genetic and environmental factors (2). The disease progresses quickly, and the abnormal B cells multiply rapidly. This can lead to a decrease in the production of normal blood cells, including red blood cells, white blood cells, and platelets (1). The diagnosis of B-cell ALL is typically confirmed through blood tests, bone marrow aspiration, and bone marrow biopsy (3). These tests help determine the percentage of abnormal B cells in the bone marrow and blood. The treatment of B-ALL usually involves a combination of chemotherapy, targeted therapy, and, in some cases, stem cell transplantation. Chemotherapy is the primary approach to eradicate the leukaemia cells, while targeted therapy may be used to specifically target certain molecules involved in the growth of B-ALL (1). The choice of the correct therapy is a critical process that helps oncologists determine the most appropriate treatment plan for individual patients and depends on the ability to stratify the patient into high risk (HR) or low/intermediate/standard risk (SR) (1, 3–5). The specific criteria and cutoff points for these categories can vary depending on the treatment protocol and the patient’s individual characteristics. The main goal is to tailor treatment to the specific characteristics of the leukaemia, taking into account factors that affect prognosis and response to therapy (3, 4). The risk stratification for B-cell ALL patients depend on different factor including:

**Age**: the age of the patient at the time of diagnosis is an important factor in risk stratification. In the case of childhood B-ALL, the age is correlated to a better outcome (6).

**White blood cell count**: the initial white blood cell count at the time of diagnosis can be indicative of the aggressiveness of the disease. High white blood cell counts may be associated with higher risk disease (7).

**Cytogenetics and genetic abnormalities**: genetic tests and cytogenetic analysis are essential in risk stratification. Specific genetic abnormalities, such as the presence of the Philadelphia chromosome (Ph+) or the presence of certain fusion genes such as *ETV6::RUNX1*, *BCR::ABL1*, or *MLL* rearrangements, can influence the risk category.

**Measurable residual disease (MRD)**: MRD refers to the small number of leukaemia cells that may remain in the body after initial treatment. Measuring MRD levels is crucial in assessing the response to therapy and predicting the risk of relapse. Lower MRD levels indicate a better prognosis.

**Response to initial treatment**: the response of the leukaemia to the initial phase of chemotherapy is also a crucial factor. Patients who achieve early, deep remission may be categorised as lower risk.

**Immunophenotype**: B-ALL can be further categorised based on the specific markers present on the leukaemia cells (e.g., CD10, CD19, CD20, CD22, CD79a). The immunophenotype can influence treatment decisions.

**Prognosis**: the prognosis for B-ALL has improved significantly in recent years, especially for children. Many patients with B-ALL can achieve long-term remission or even be considered cured with the appropriate treatment. The outlook for adults with B-cell ALL varies, and it depends on factors such as age, overall health, and specific genetic features of the leukaemia.

Advances in medical research and treatment have significantly improved the outcomes for patients with B-ALL, making it a treatable condition in many cases. Once risk stratification is complete, treatment plans can be tailored to provide the most appropriate therapy for the patient’s risk group and clinical features. Patients in higher risk categories may receive more aggressive treatment, including stem cell transplantation, to improve their chances of achieving remission and long-term survival (8, 9). Lower risk patients may receive less-intensive therapy to minimize side effects. It is important to note that risk stratification is a dynamic process, and treatment plans may be adjusted based on the patient’s response to therapy and evolving risk factors throughout the course of treatment (9, 10). Medical professionals specialising in leukaemia treatment use a multidisciplinary approach to ensure the best care for each patient. It is therefore necessary to identify methods to categorise the risk of patients with B-ALL, which are as personalised and objective as possible, in order to avoid the use of ineffective therapies that may lead to relapse.

In our previous work (11), we first identified LINC00958 (also known as BLACAT2) in paediatric B-ALL patients. This lncRNA has already been associated with neoplastic progression in both bladder cancer (12), gastric cancer (13), and leukaemia (14). LINC00958 is able to discriminate B-ALL from healthy subjects and T-ALL and to significantly stratify standard/medium risk patients from HR patients. In addition, the expression levels of LINC00958 can identify, already at diagnosis, patients who will relapse within 3 years after leukaemia diagnosis. Furthermore, preliminary functional studies on a B-ALL cell system show that LINC00958 could be involved in the regulation of the cell cycle of leukaemic cells by determining the transition from S-phase to G2-M phase, making it a potential therapeutic target for the treatment of paediatric B-ALL. This information may be crucial in developing new diagnostic approaches for childhood of B-ALL, so that the medical team managing the B-ALL patient can choose the most appropriate therapy to ensure the best possible outcome of the paediatric patient.

## Methods

### Study population

Patients were enrolled following the guidelines reported in the study approved by the local ethical committees of the IRCCS SYNLAB SDN, in line with the Helsinki declaration [Ethical Committee IRCCS Pascale, Naples, Italy—protocol number 5/19 of the 19 June 2019, and the AORN Santobono-Pausilipon (Ethical Committee Cardarelli/Pausilion, Naples Italy—protocol number 07/20 of 3 June 2020)]. All participants provided informed assent through informed consent signed by both parents. The clinical data of enrolled patients are reported in **Table 1**.

**Table 1:** Clinical characteristics of childhood acute lymphoblastic leukaemia patients.

	B-ALL	T-ALL
**Number (%)**	33 (70,6)	10 (29,4)
**Median Age at diagnosis (range)**	6,6 years (range 1 month–17 years)	8 years (range 2–17 years)
**Sex (%)**	9 female (37,5) 24 male (62,5)	4 female (40) 6 male (60)
**Race (%)**	33 Caucasian	7 Caucasian (70) 3 non-Caucasian (30)
**hyperleukocytosis at diagnosis (%)**	7 (21) >100,000 µl 26 (79) < 100,000 µl	7 (70) >100,000 µl 3 (30) < 100,000 µl
**Cytogenetics (%)**	19 (57,6) not available 8 (24,2) normal 2 (6) *t*(9;22) positive 2 (6) (11q23 riarrangement) 2 (6) complex cariotype	2 (20) normal 4 (40) not available 2 (20) hyperdiploid 2 (20) complex cariotype
**Molecular biology (%)**	2 (6) BCR ABL 1 (3) KMT2A/MLLT1 1 (3) MLL/AF4 1 (3) TEL/AML1 28 (84,8) negative for rearrangement	2 (20) SIL/TAL, 8 (80) negative
**EGIL classification (%)**	3 EGIL B I (8,3) 27 EGIL B II (83,3) 3 EGIL B III (8,3)	1 EGIL T I/II (10) 8 EGIL T–III (80) 1 EGIL T–IV (10)
**Extramedullary status**	0 estramedullary disease	1 SNC 3 and testis 1 SNC2 1 mediastinum bulky
**Risk**	18 non-high risk 15 high risk	2 non-high risk 8 high risk
**Relapse**	16 relapse 17 no relapse	1 relapse 9 no relapse
**Alive**	31 alive 1 death 1 lost in follow-up	1 death 8 alive 1 lost follow-up

B-ALL, acute lymphoblastic leukaemia of B-cell patients. T-ALL, acute lymphoblastic leukaemia of T-cell patients.

### Patients sample and cell line

Bone marrow mononuclear cells (BM–MNCs) of patients and cord blood mononuclear cells used as healthy controls were recovered from IRCCS SYNLAB SDN biobank (15). Authenticated human leukaemia B-cell line SEM was grown in IMDM (Iscove’s Modified Dulbecco’s Medium), supplemented with heat-inactivated fetal bovine serum, 100 U/ml penicillin, 100 mg/ml streptomycin, 1% l-glutamine at 37°C in a 5% CO_2_ atmosphere. LINC00958 silencing in SEM cell line was conducted by electroporation by using the Neon™ Transfection System (Invitrogen, Thermo Fisher Scientific, US) and dicer-substrate short-interfering RNAs (DsiRNAs) and TriFECTa^®^ Kits (Integrated DNA Technologies, Coralville, IA, USA). Cells were collected 48h after transfection and subjected to further analysis.

### RNA extraction and RT-PCR experiments

RNA extraction and reverse transcription polymerase chain reaction (RT-PCR) experiments were performed as previously described (16). The RPS18 gene was used as housekeeping. The oligonucleotides used for RT-PCR were reported in **Table 2**.

**Table 2:** Oligonucleotide for RT-PCR experiments.

Gene name	Forward	Reverse
**RPS18**	5′-CGATGGGCGGCGGAAAATA-3’	5′-CTGCTTTCCTCAACACCACA-3’
**LINC00958**	5’- TGCAGCAAGATAGCTCCAGG-3’	5’-CCTGGCGTCTGTGTAGTGTT-3’
**CyclinA**	5′-AAATGGGCAGTACAGGAGGA-3′	5′- CCACAGTCAGGGAGTGCTTT-3′
**CyclinB**	5′-CATGGTGCACTTTCCTCCTT-3′	5′ AGGTAATGTTGTAGAGTTGGTGTCC-3′
**CyclinE**	5′-GGCCAAAATCGACAGGAC-3′	5′-GGGTCTGCACAGACTGCAT-3′
**CyclinD**	5′-GCTGTGCATCTACACCGACA-3	5′-TTGAGCTTGTTCACCAGGAG-3′
**p53**	CCC CTC CAT CCT TTC TTC TC	ATG AGC CAG ATC AGG GAC TG

### Functional studies

Functional studies were performed on SEM and RS4;11 cell lines treated for 48h with DsiRNAs against LINC00958. DNA-Prep Reagents kit (607055, Beckman Coulter, Brea, CA, USA) was used to analyse the cell cycle of SCRAMBLE and DsiRNA-LINC00958–treated SEM cells using a minimum of 10,000 single-cell events recorded. The percentage of G1, S, and G2/M phases was calculated using the Michael Fox algorithm. Afterward, the analysis of the cell cycle was conducted using Kaluza Analysis Software 2.1 (Beckman Coulter). ATP lite™ (6016943, PerkinElmer, Waltham, MA, USA) was used to detect the cell viability. ATP signal was measured using the Victor Nivo plate reader (PerkinElmer, Waltham, MA, USA). Cell cycle and ATP lite were conducted in triplicate with similar results. Western blot of P53 protein was conducted using 50 µg of cell whole cell extract derived from SEM cells electroporated with siRNA against LINC00958 and scramble control using the following antibodies:

- anti-p53 (#sc-126, Santa Cruz Biotechnology, Dallas, TX, USA)

- anti-vinculin (VLN01, Life Technologies, USA)

WB was acquired using the ChemiDoc imaging system (Bio-Rad laboratories, USA) coupled with Image Lab software.

### Statistical analyses

Statistical analyses were performed using GraphPad Prism Version 9. Mann–Whitney *t*-test was used to assess the statistical significance of comparisons. *P* < 0.05 were considered statistically significant (*P < 0.05, **P < 0.01, ***P < 0.001). For the functional experiments, error bars represent mean ± SD of, at minimum, three independent experiments.

### Pathway activity score correlation

Pathway activity score (PAS) for Reactome pathway database was calculated with testSctpa (https://github.com/zgyaru/testSctpa) in every childhood ALL samples from St. Jude Children’s Research Hospital database. The resulting scores were correlated with LINC00958 transcript expression using Pearson correlation. Plot representation was obtained with R package ggplot2.

## Results

### LINC00958 is specifically expressed in paediatric B-cell acute lymphoblastic leukaemia

**Figure 1:**
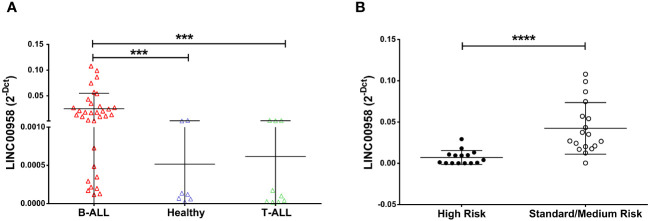
Expression level of LINC00958 in a cohort of paediatric B-ALL (red triangle *n* = 27), T-ALL (green triangles *n* = 10) and healthy donors (blue triangles *n* = 7) **(A)** and in the B-ALL patients separated in high-risk patients (fill black circles *n* = 10) and standard/medium risk patients (empty circles *n* = 17) **(B)**. Expression levels were plotted according to the relative expression (2-ΔCt method) of LINC00958. ***p < 0.001; ****p < 0.0001; Mann–Whitney *t*-test.

Recent studies (11) already identified LINC00958 as a possible B-ALL biomarker. To deeply investigate LINC00958 diagnostic potential, we evaluated the expression level of LINC00958 in a further group of paediatric B-ALL patients, T-cell acute lymphoblastic leukaemia (T-ALL), and healthy donors. LINC00958 expression resulted to be significantly higher in B-ALL patients respect to the T-ALL (*p* = 0.0001) patients and healthy donors (*p* = 0.0003) (**Figure 1A**). Its expression was also evaluated in a larger case series of paediatric patients with different types of acute lymphoblastic leukaemia from the Saint Jude Children’s Research Hospital database (https://platform.stjude.cloud/data/cohorts/paediatric-cancer) (17). As shown in **Supplementary Figure S1**, also in this dataset, LINC00958 was significantly more expressed in B-ALL than in T-ALL, suggesting that it may be reflective of B-cell versus T-cell biology. Afterwards, B-ALL patients from our case study were classified as HR and SR (considering in this cohort all the non-HR B-ALL patients) or based on age, white blasts count, and central nervous system status (patients clinical information are reported in **Table 1** in the Method section). It was investigated if, in the context of pathology, LINC00958 also correlated with a specific risk category. To this end, the expression of LINC00958 was analysed by real-time PCR in samples from SR and HR patients (**Figure 1B**), and it surprisingly emerged that LINC00958 was significantly over-expressed in SR patients respect to the HR patients (*p* < 0.0001). To evaluate the clinical implications of LINC00958, its expression was correlated with different patients’ clinical information. No significant correlations were identified between LINC00958 expression levels with either the age of paediatric patients or the white blood counts at diagnosis or the percentage of blasts at diagnosis evaluated by flow cytometry (data not shown).

### LINC00958 is higher in relapsed B-ALL patients

**Figure 2:**
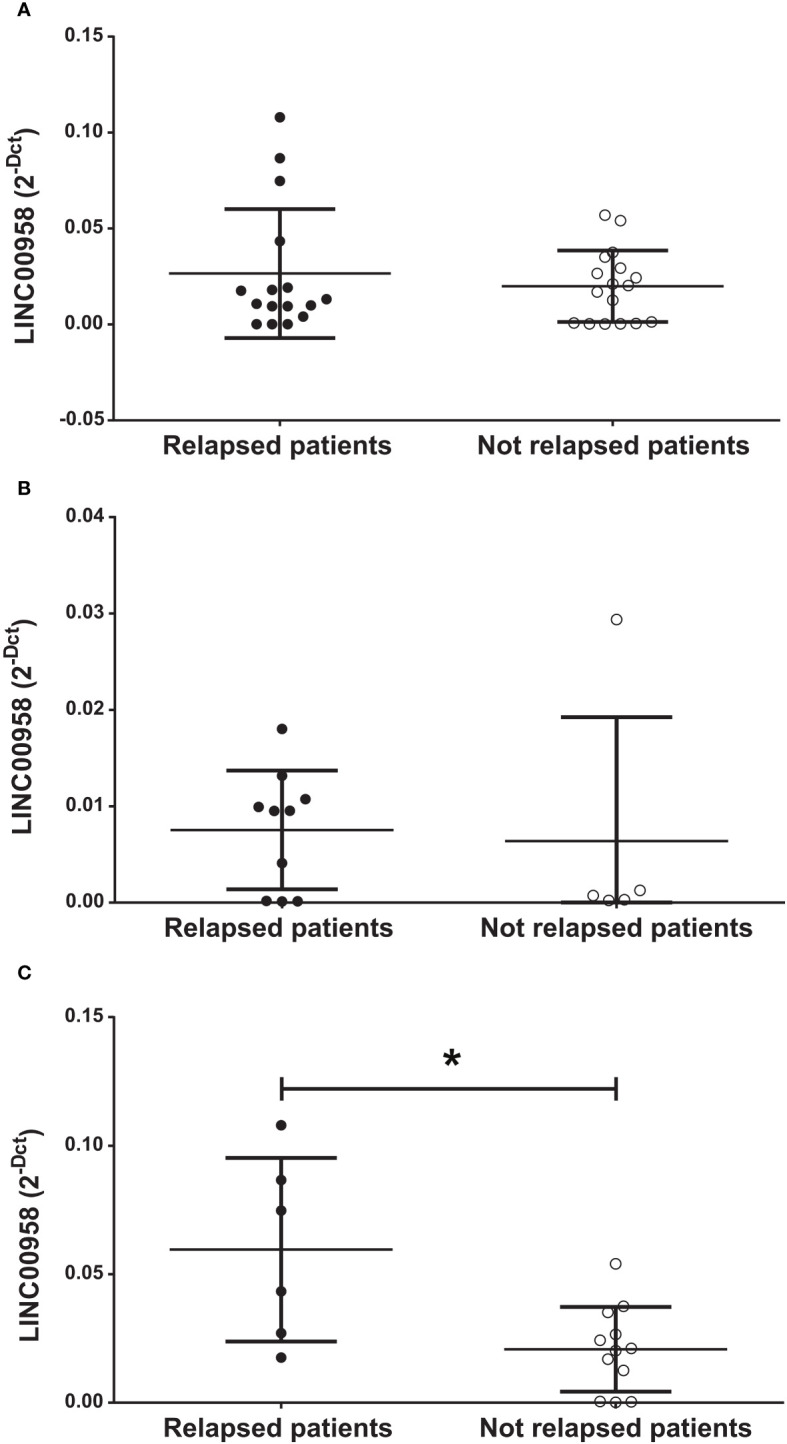
Expression level of LINC00958 in relapsed (fill black circles) and non-relapsed (empty circles) in the entire cohort of paediatric B-ALL patients **(A)**, in high-risk patients **(B)** and in standard-risk patients **(C)**. *p < 0.05. n.s. = not significant. Mann–Whitney *t*-test. Error bars represent the standard deviation relapsing patients were considered at 3 years after the diagnosis of B-ALL.

To gain a more comprehensive insight into the possible prognostic role of LINC00958 for B-ALL patients who relapsed, we evaluated if LINC00958 levels were predictive of relapse within 3 years after the diagnosis. As shown in **Figures 2A, B**, there were no significant differences between patients who relapsed versus those who did not relapse when considering all the B-ALL patients in our cohort (**Figure 2A**). Nonetheless, when separating HR patients from SR patients, we found that, while there continued to be no difference in HR patients (**Figure 2B**), LINC00958 expression levels in SR childhood patients is significantly higher in the relapsed patients respect to the non-relapsed patients (**Figure 2C**, *p* = 0.0182).

### Silencing of LINC00958 results in decreased proliferation in B-ALL cell line model

**Figure 3:**
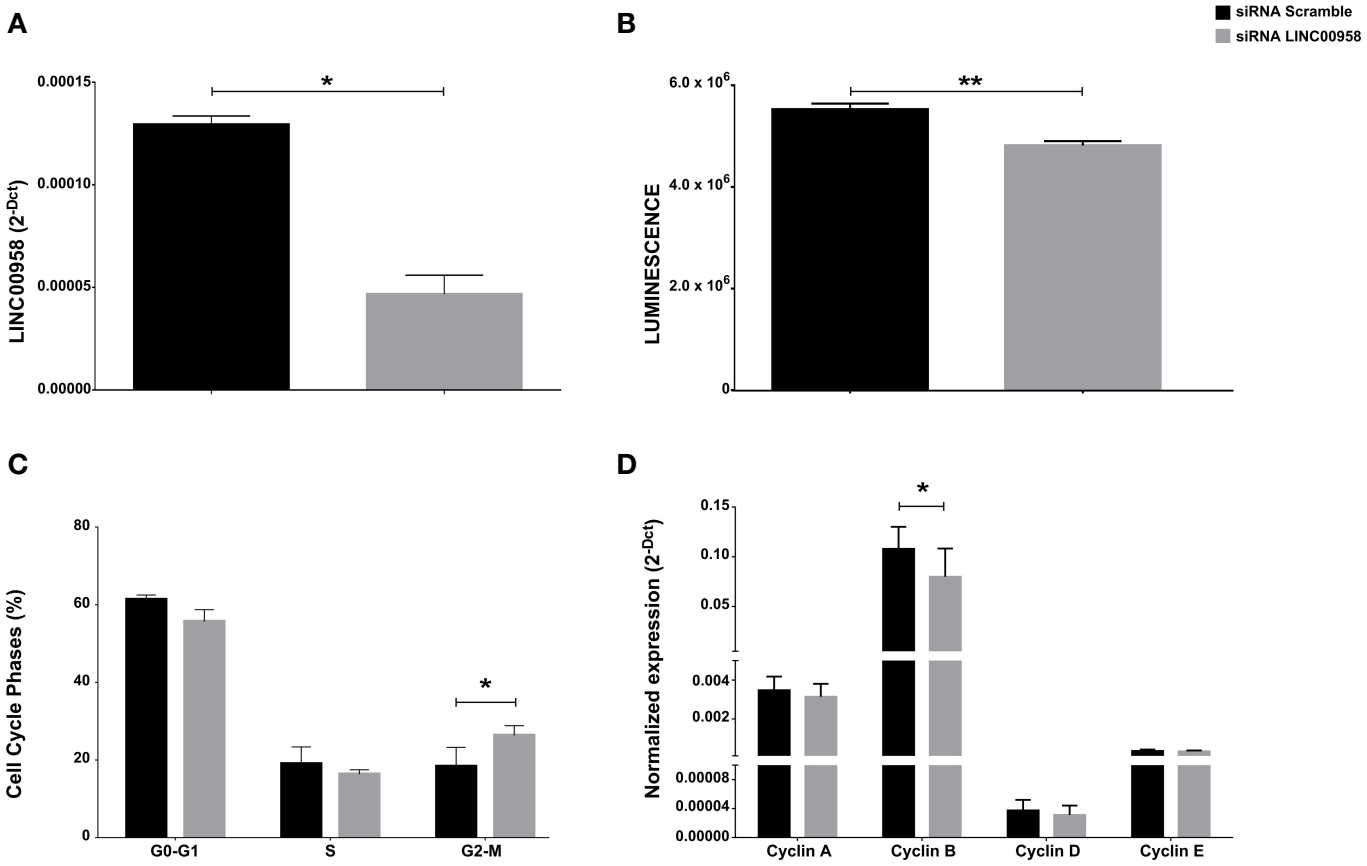
**(A)** LINC00958 expression level in siRNA scramble–treated SEM (black bar) and specific siRNA against LINC00958-treated SEM (grey bar) after 48h of incubation. Error bars represent the SD of three independent experiments. **(B)** ATPlite assay of siRNA scramble–treated SEM (black bar) and siRNA LINC00958 treated SEM (grey bar) after 48h of incubation. Error bars represent the SD of six independent experiments. **(C)** Cell cycle phases of siRNA scramble–treated SEM (black bar) and siRNA LINC00958–treated SEM (grey bar) after 48h of incubation. The percentages of cells in the G0–G1, S, and G2–M phases after 48h of active growth were reported as the mean values of three independent experiments ± SD. **(D)** mRNA expression levels of cyclins in siRNA scramble-treated SEM (black bar) and siRNA LINC00958–treated SEM (grey bar) after 48h of incubation. Relative expression was determined using the 2−ΔCt method. Relative expression of cyclins is shown as mean ± SD of three technical independent experiments. *p < 0.05. Mann–Whitney *t*-test. Error bars represent the standard deviation. **p-value < 0,001.

**Figure 4:**
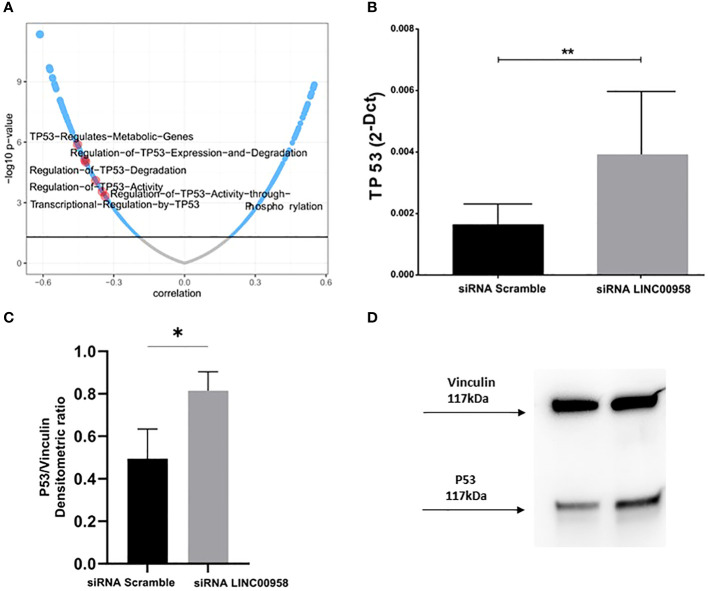
**(A)** LINC00958 expression correlation with REACTOME PATHWAYS scores in ALL sample database from Saint Jude’s Children Hospital. Terms involving the regulation of TP53 are highlighted in red. **(B)** Real Time PCR showing the TP53 expression levels in SEM cells electroporated with siRNA against LINC00958 and scramble control. **p < 0.01. **(C)** Western blot of P53 protein of SEM cells electroporated with siRNA against LINC00958 and scramble control. **(D)** Expression levels of P53 protein evaluated from the densitometry of bands normalised towards the corresponding densitometry of Vinculin bands. Mann–Whitney *t*-test. Error bars represent the standard deviation of three independent experiments. *p-value<0,05.

As a step following the clinical evaluations on the possible prognostic role of LINC00958 in acute paediatric lymphoblastic leukaemia, a model system for B-ALL was used to investigate any potential functional consequences arising from the silencing of LINC00958. A human pro-B-ALL cell line, the SEM cell line, was electroporated with pre-validated siRNA that individually target LINC00958 RNA molecule and non-specific control siRNA (siRNA scramble). Effective silencing was ascertained by real-time PCR 48h after electroporation (**Figure 3A**). A luminescent cell viability assay, conducted 72h after electroporation, showed a significant decrease of cell proliferation in LINC00958 knockdown sample (**Figure 3B**). A cytofluorimetric analysis of the cell cycle, 72h after electroporation, showed an arrest at G2-M phase in siRNA transfected in comparison to scrambled control-transfected SEM cells (**Figure 3C**; **Supplementary Figure S2**). To deeply investigate the cell cycle modulation observed in the LINC00958 knockdown cells, we evaluated the expression levels of cyclin genes, as they play a pivotal role in cell cycle regulation. Cyclins expression was evaluated by real-time PCR 72h after electroporation. We observed a significant difference in expression for cyclin B in LINC00958 knockdown cells (**Figure 3D**). To further investigate a possible role for LINC00958 in cell cycle and proliferation, we calculated the pathway activity score (PAS) for Reactome pathway database of every childhood ALL samples from St. Jude Children’s Research Hospital database. The resulting scores were correlated with LINC00958 transcript expression values in the same sample. We noticed a redundant enrichment of P53 activity on cell-proliferation-related pathways among the terms with highest inverse correlation and significance values (**Figure 4A**). We also registered a significant upregulation of P53 in LINC00958 knockdown SEM cells (**Figures 4B–D**), confirming an anti-correlation between the expression of LINC00958 and P53. These data were replicated and confirmed in a second B-ALL cell line model, with similar results (**Supplementary Figure S3**).

## Discussion

ALL is the most common oncological malignancy in children, accounting for one-third of all childhood cancers and the most represented immunophenotype is B-lineage. B-cell acute lymphoblastic leukaemia results from clonal proliferation of abnormal B-lymphoblasts in bone marrow. B-ALL is classified into different risk subgroups, standard and high, based on genetic, biological, and clinical features such as sex and age at diagnosis, immunophenotypic, cytogenetic, molecular, and early medullar response to induction therapy (18, 19). Among the SR patients, the rate of early and late relapse was approximately 35% and 5%, respectively, compared to 50% and 1% among the HR patients (1, 20–22). One important issue in optimising outcomes for ALL patients lies is the limited ability to predict likelihood of relapse. We have previously identified a lncRNA signature discriminating paediatric B-ALL and T-ALL from healthy subjects (11). We selected lncRNAs that have never been associated with childhood ALL, among them LINC00958, also named bladder cancer-associated transcript 2 (BLACAT2), that was found markedly upregulated in lymph node-metastatic bladder cancer (12). In this work, the role of LINC00958 in B-ALL was investigated. As already described, LINC00958 gene discriminates between B-ALL and T-ALL, being over-expressed in B-ALL. Here, aside from validating the role of LINC00958 as a molecular marker of B-ALL in a larger cohort of patients, we further show that its higher expression is also associated with B-ALL SR type. Hence, correlating the clinical history of SR patients with LINC00958 expression, a very interesting finding emerged: from the outset, a pronounced upregulation of LINC00958 is discernible in patients who subsequently experience disease relapse. This evidence underscores a direct correlation between the expression of LINC00958 and an elevated susceptibility to relapse, suggesting LINC00958 as a potential novel genetic biomarker that could enhance the precision of risk stratification and ultimately ameliorate clinical outcomes. Monitoring LINC00958 levels holds promise for optimising relapse prevention strategies. Furthermore, given the infrequent occurrence of relapse among SR patients, the upregulation of LINC00958 in all sixteen examined cases of relapse assumes heightened significance within the dataset. Silencing LINC00958 in a cell-culture model system for B-ALL results in a decrease in proliferation in according with the evidence that LINC00958 acts as an oncogene in various types of cancers (12, 13, 23). This finding is associated with a G2-phase arrest observed in cell-cycle analysis and downregulation of cyclin B. Concurrently, our analyses show a strong inverse correlation between the expression levels of LINC00958 and P53 in ALL. The observed reduction in cell proliferation can be attributed to the concurrent upregulation of P53 at RNA and protein level. P53 is a tumour suppressor gene that plays a pivotal role in regulating cell growth and preventing the proliferation of damaged or abnormal cells. When TP53 is upregulated, it exerts its inhibitory influence on the cell cycle, leading to a deceleration in cell proliferation (24–26). Our findings can be explained through the established P53-dependent G2 arrest mechanism, a well-documented phenomenon extensively described in the scientific literature (27, 28). The G2/M transition is governed by the regulation of cyclin-dependent kinase CDC2 and its critical positive regulatory subunit, cyclin B. The kinase activity of the CDC2-cyclin B complex, along with the levels of cyclin B, undergoes cyclic fluctuations throughout the cell cycle. Elevated expression of TP53 leads to the suppression of cyclin B/CDC2 kinase activation, resulting in the subsequent arrest of cells in the G2 phase (26). In response to DNA damage, elevated expression of P53 inhibits the activities of cyclin B and CDC2, resulting in an increased mitosis and subsequent arrest of cells in the G2 phase (26, 29, 30). This observation allows us to hypothesise a role of LINC00958 in the G2 DNA damage checkpoint. However, it remains to be clarified how LINC00958 exerts its influence, particularly in the G2 phase of the cell cycle and in the presence of DNA damage.

In conclusion, our study has demonstrated the specific association of LINC00958 with the SR category in childhood B-cell acute lymphoblastic leukaemia. It is important to note that our study has some limitations related to the low-sample size. This is certainly linked to the fact that this study has only one enrolling centre and to the fact that the disease is rare (acute lymphoblastic leukaemia affects about eight children per 100,000). Future studies will be conducted on larger case series, with the involvement of other recruiting centres, to consider using LINC00958 in the clinical management of paediatric leukaemia patients. Notably, LINC00958 exhibits a particularly strong correlation with the risk of disease relapse, even in cases initially classified as SR based on early diagnosis. Functionally, LINC00958 appears to play a significant role in promoting cell proliferation and perturbing cell cycle regulation, potentially through P53 activity. These findings shed light on the intricate and nuanced factors contributing to leukaemia risk stratification and disease progression, highlighting LINC00958 as a potential biomarker and therapeutic target in B-cell acute lymphoblastic leukaemia.

## Conclusions

Our findings provide crucial insights into the potential utility of LINC00958 as a genetic diagnostic and prognostic marker in the context of B-cell acute lymphoblastic leukaemia. This holds promise for enhancing the precision of relapse prediction, particularly among paediatric patients conventionally categorised as SR. Such advancement in risk stratification could pave the way for proactive therapeutic interventions aimed at mitigating the risk of disease recurrence in this vulnerable patient population.

## Data availability statement

The raw data supporting the conclusions of this article will be made available by the authors, without undue reservation.

## Ethics statement

The studies involving humans were approved by Ethical Committee IRCCS Pascale, Naples, Italy—protocol number 5/19 of the 19 June 2019, and the AORN Santobono-Pausilipon (Ethical Committee Cardarelli/Pausilion, Naples Italy—protocol number 07/20 of 3 June 2020. The studies were conducted in accordance with the local legislation and institutional requirements. Written informed consent for participation in this study was provided by the participants’ legal guardians/next of kin.

## Author contributions

FA: Data curation, Methodology, Writing – original draft. LB: Formal analysis, Investigation, Methodology, Writing – original draft, Writing – review & editing. ML: Data curation, Formal analysis, Writing – review & editing. PM: Conceptualization, Writing – review & editing. AC: Data curation, Formal analysis, Writing – review & editing. GB: Conceptualization, Data curation, Formal analysis, Writing – review & editing. AM: Conceptualization, Formal analysis, Writing – review & editing. RP: Conceptualization, Formal analysis, Writing – review & editing. MS: Project administration, Supervision, Writing - review & editing. IS: Funding acquisition, Writing – review & editing. GS: Data curation, Project administration, Supervision, Validation, Writing – original draft, Writing – review & editing.

## References

[1] PDQ Pediatric Treatment Editorial Board. Childhood acute lymphoblastic leukemia treatment (PDQ®): health professional version. In: PDQ Cancer Information Summaries. National Cancer Institute (US, Bethesda (MD (2002). Available at: http://www.ncbi.nlm.nih.gov/books/NBK65763/. 26389206

[2] Yeung DTO Osborn MP White DL. B-cell acute lymphoblastic leukaemia: recent discoveries in molecular pathology, their prognostic significance, and a review of the current classification. Br J Haematol. (2022) 197:13–27. doi: 10.1111/bjh.17879 34747016

[3] Chiaretti S Zini G Bassan R. Diagnosis and subclassification of acute lymphoblastic leukemia. Mediterr J Hematol Infect Dis. (2014) 6:e2014073. doi: 10.4084/mjhid.2014.073 25408859 PMC4235437

[4] Vrooman LM Blonquist TM Harris MH Stevenson KE Place AE Hunt SK. Refining risk classification in childhood B acute lymphoblastic leukemia: results of DFCI ALL Consortium Protocol 05–001. Blood Adv. (2018) 2:1449–58. doi: 10.1182/bloodadvances.2018016584 PMC6020806 29941458

[5] Bassan R Pavoni C Intermesoli T Spinelli O Tosi M Audisio E. Updated risk-oriented strategy for acute lymphoblastic leukemia in adult patients 18–65 years: NILG ALL 10/07. Blood Cancer J. (2020) 10:1–14. doi: 10.1038/s41408-020-00383-2 33188164 PMC7666128

[6] Ahmed AM Al-Trabolsi H Bayoumy M Abosoudah I Yassin F. Improved outcomes of childhood acute lymphoblastic leukemia: A retrospective single center study in Saudi Arabia. Asian Pac J Cancer Prev APJCP. (2019) 20:3391–8. doi: 10.31557/APJCP.2019.20.11.3391 PMC7063019 31759364

[7] Teachey DT Pui CH. Comparative features and outcomes between paediatric T-cell and B-cell acute lymphoblastic leukaemia. Lancet Oncol. (2019) 20:e142–54. doi: 10.1016/S1470-2045(19)30031-2 PMC9233195 30842058

[8] Bhojwani D Howard SC Pui CH. High-risk childhood acute lymphoblastic leukemia. Clin Lymphoma Myeloma. (2009) 9:S222. doi: 10.3816/CLM.2009.s.016 19778845 PMC2814411

[9] DeAngelo DJ Jabbour E Advani A. Recent advances in managing acute lymphoblastic leukemia. Am Soc Clin Oncol Educ Book Am Soc Clin Oncol Annu Meet. (2020) 40:330–42. doi: 10.1200/EDBK_280175 32421447

[10] Oh BLZ Lee SHR Yeoh AEJ. Curing the curable: Managing low-risk acute lymphoblastic leukemia in resource limited countries. J Clin Med. (2021) 10:4728. doi: 10.3390/jcm10204728 34682851 PMC8540602

[11] Buono L Iside C De Matteo A Stellato P Beneduce G de Vera d’Aragona RP. Specific lncRNA signatures discriminate childhood acute leukaemias: a pilot study. Cancer Cell Int. (2022) 22:373. doi: 10.1186/s12935-022-02789-3 36451206 PMC9710039

[12] He W Zhong G Jiang N Wang B Fan X Chen C. Long noncoding RNA BLACAT2 promotes bladder cancer-associated lymphangiogenesis and lymphatic metastasis. J Clin Invest. (2018) 128:861–75. doi: 10.1172/JCI96218 PMC5785244 29355840

[13] Hu H Kong Q Huang XX Zhang HR Hu KF Jing Y. Longnon-coding RNA BLACAT2 promotes gastric cancer progression via the miR-193b-5p/METTL3 pathway. J Cancer. (2021) 12:3209–21. doi: 10.7150/jca.50403 PMC8100803 33976730

[14] Fernando TR Rodriguez-Malave NI Waters EV Yan W Casero D Basso G. LncRNA expression discriminates karyotype and predicts survival in B-lymphoblastic leukemia. Mol Cancer Res MCR. (2015) 13:839–51. doi: 10.1158/1541-7786.MCR-15-0006-T PMC4433429 25681502

[15] Mirabelli P Incoronato M Coppola L Infante T Parente CA Nicolai E. SDN biobank: bioresource of human samples associated with functional and/or morphological bioimaging results for the study of oncological, cardiological, neurological, and metabolic diseases. Open J Bioresour. (2017) 4:2. doi: 10.5334/ojb.26

[16] Buono L Iside C Pecoraro G De Matteo A Beneduce G Penta de Vera d’Aragona R. A comprehensive analysis of the expression profiles of KCTD proteins in acute lymphoblastic leukemia: Evidence of selective expression of KCTD1 in T-ALL. J Clin Med. (2023) 12:3669. doi: 10.3390/jcm12113669 37297863 PMC10253327

[17] McLeod C Gout A Zhou X Rahbarinia D Thrasher A Newman S. St. Jude cloud—a pediatric cancer genomic data sharing ecosyste. Cancer Discov. (2021) 11(5):1082–99. doi: 10.1158/2159-8290.CD-20-1230 PMC8102307 33408242

[18] Moorman AV. New and emerging prognostic and predictive genetic biomarkers in B-cell precursor acute lymphoblastic leukemia. Haematologica. (2016) 101:407–16. doi: 10.3324/haematol.2015.141101 PMC5004393 27033238

[19] Sun W Malvar J Sposto R Verma A Wilkes JJ Dennis R. Outcome of children with multiply relapsed B-cell acute lymphoblastic leukemia: a therapeutic advances in childhood leukemia & lymphoma study. Leukemia. (2018) 32:2316–25. doi: 10.1038/s41375-018-0094-0 PMC6224404 29728694

[20] Oskarsson T Söderhäll S Arvidson J Forestier E Montgomery S Bottai M. Relapsed childhood acute lymphoblastic leukemia in the Nordic countries: prognostic factors, treatment and outcome. Haematologica. (2016) 101:68–76. doi: 10.3324/haematol.2015.131680 26494838 PMC4697893

[21] Bhojwani D Pui CH. Relapsed childhood acute lymphoblastic leukaemia. Lancet Oncol. (2013) 14:e205–217. doi: 10.1016/S1470-2045(12)70580-6 23639321

[22] Tuong PN Kiem Hao T Kim Hoa NT. Relapsed childhood acute lymphoblastic leukemia: A single-institution experience. Cureus. (2020) 12:e9238. doi: 10.7759/cureus.9238 32821584 PMC7430696

[23] Zhang C Wang R Li M Yang Q. Long non−coding RNA BLACAT2/miR−378a−3p/YY1 feedback loop promotes the proliferation, migration and invasion of uterine corpus endometrial carcinoma. Oncol Rep. (2023) 49:108. doi: 10.3892/or 37052291 PMC10152453

[24] Marei HE Althani A Afifi N Hasan A Caceci T Pozzoli G. p53 signaling in cancer progression and therapy. Cancer Cell Int. (2021) 21:703. doi: 10.1186/s12935-021-02396-8 34952583 PMC8709944

[25] Engeland K. Cell cycle regulation: p53-p21-RB signaling. Cell Death Differ. (2022) 29:946–60. doi: 10.1038/s41418-022-00988-z PMC9090780 35361964

[26] Taylor WR Stark GR. Regulation of the G2/M transition by p53. Oncogene. (2001) 20:1803–15. doi: 10.1038/sj.onc.1204252 11313928

[27] Agirre X Novo FJ Calasanz MJ Larráyoz MJ Lahortiga I Valgañón M. TP53 is frequently altered by methylation, mutation, and/or deletion in acute lymphoblastic leukaemia. Mol Carcinog. (2003) 38:201–8. doi: 10.1002/mc.10159 14639659

[28] Coffey RNT Watson RWG O’Neill AJ Mc Eleny K Fitzpatrick JM. Androgen-mediated resistance to apoptosis. Prostate. (2002) 53:300–9. doi: 10.1002/pros.10159 12430141

[29] Agarwal ML Agarwal A Taylor WR Stark GR. p53 controls both the G2/M and the G1 cell cycle checkpoints and mediates reversible growth arrest in human fibroblasts. Proc Natl Acad Sci U S A. (1995) 92:8493–7. doi: 10.1073/pnas.92.18.8493 PMC41183 7667317

[30] Flatt PM Tang LJ Scatena CD Szak ST Pietenpol JA. p53 regulation of G(2) checkpoint is retinoblastoma protein dependent. Mol Cell Biol. (2000) 20:4210–23. doi: 10.1128/MCB.20.12.4210-4223.2000 PMC85790 10825186

