# Supplementary material for: LINC00958 as new diagnostic and prognostic biomarker of childhood acute lymphoblastic leukaemia of B cells
Source: Front Oncol. 2024 May 31;14:1388154. doi: 10.3389/fonc.2024.1388154 (PMC11176504; doi:10.3389/fonc.2024.1388154)
Supplement: Supplementary file 1 [file DataSheet_1.pdf]

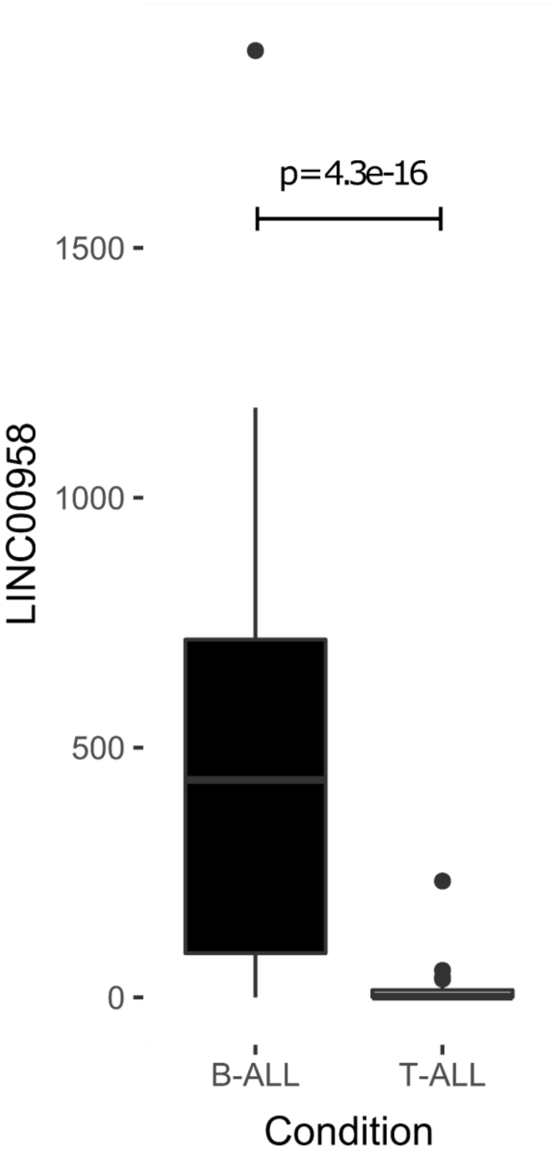

Supplementary Figure 1. LINC00958 expression levels using RNA-seq normalize count data from the St. Jude Children's Research Hospital database. B-ALL patients: n = 80, median age: 15 years. T-ALL patients: n = 25, median age: 9 years.

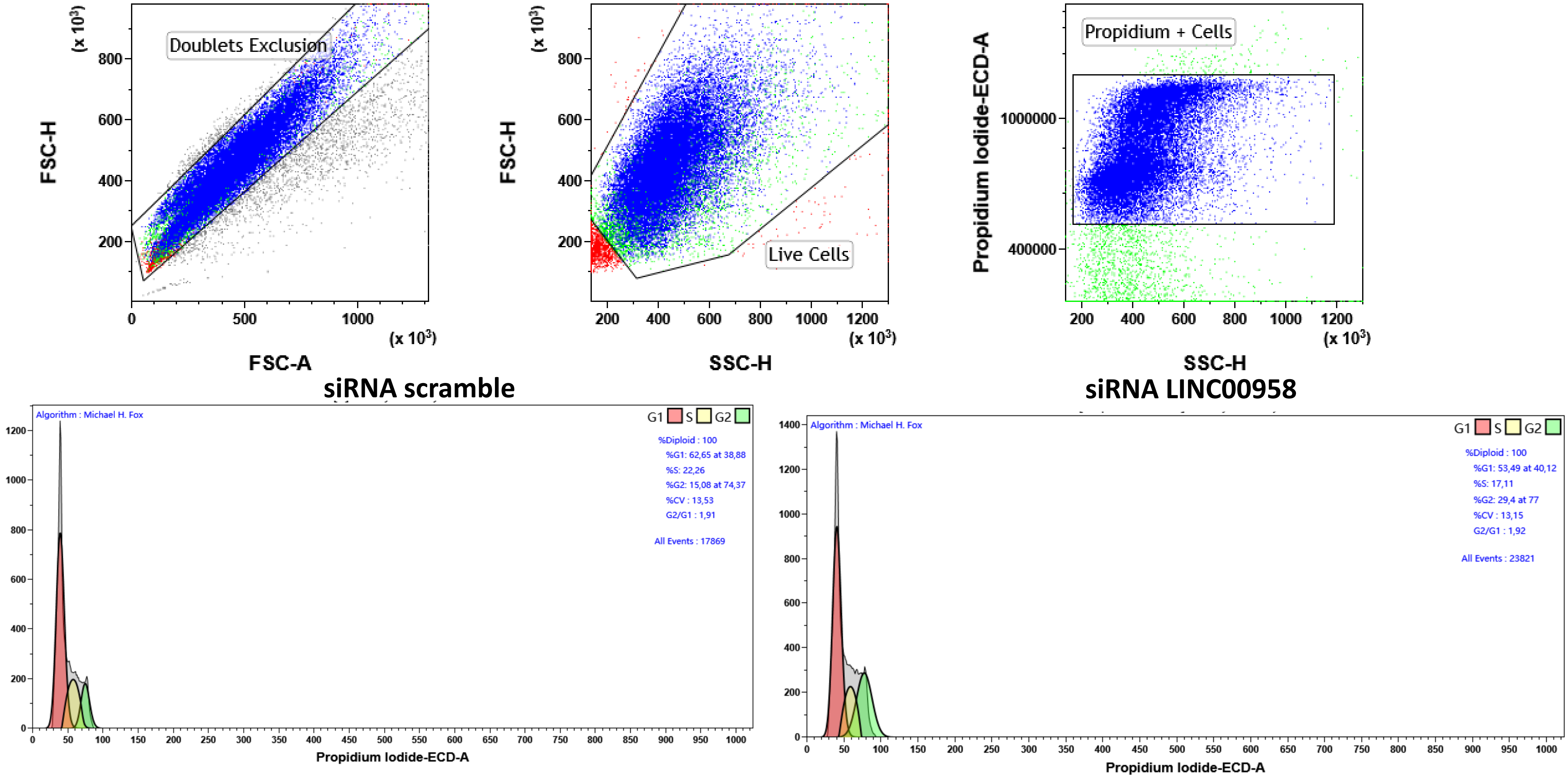

Supplementary Figure 2. Gating strategy for the detection of cell cycle phases. Doublets exclusion (left panel), live cells (middle panel), Propidium Iodide positive cells (right panel). Bottom panels report a representative flow cytometry analysis of the cell cycle distribution using Micheal H. Fox algoritm.

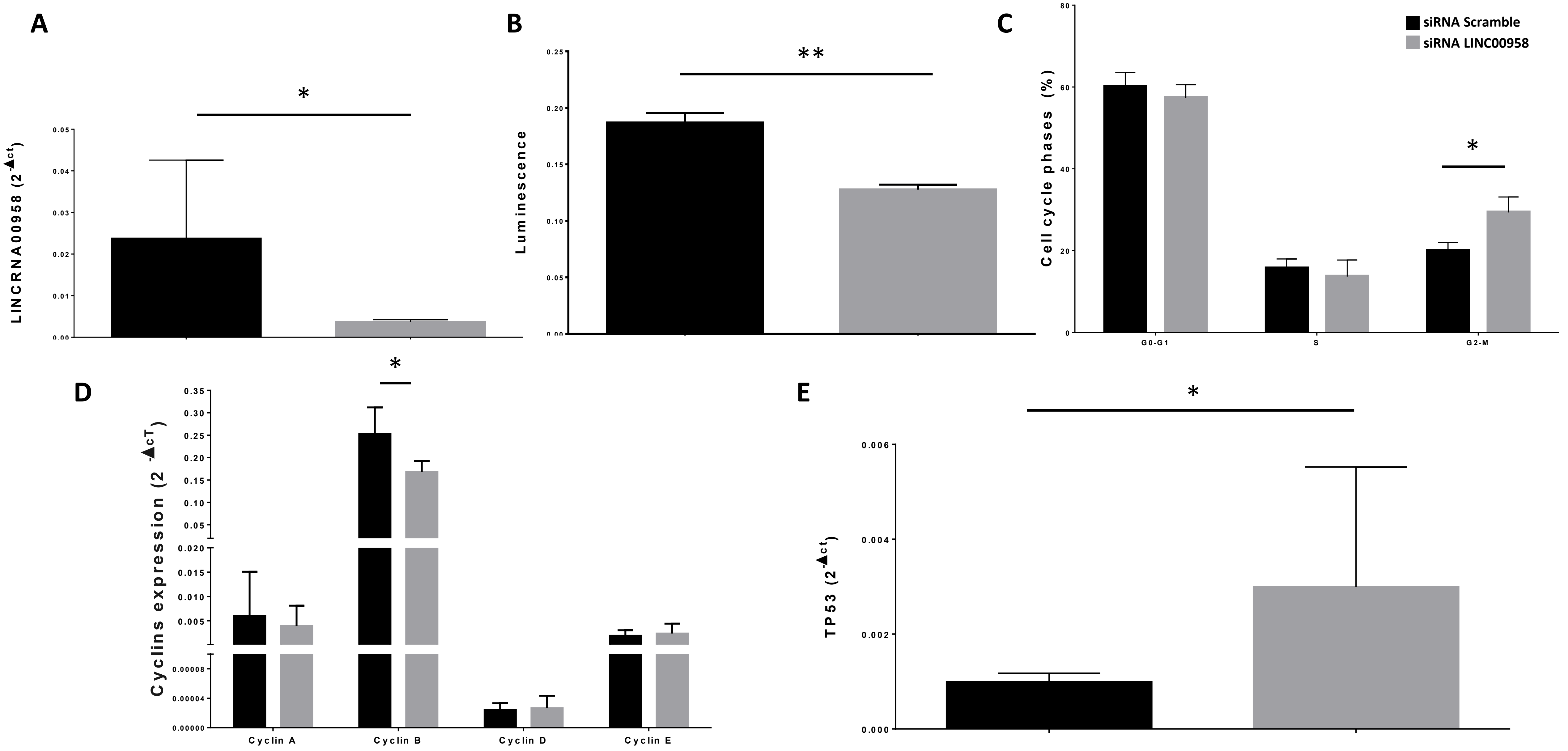

Supplementary Figure 3. (A) LINC00958 expression level in siRNA scramble treated RS4;11 (black bar) and specific siRNA against LINC00958 treated RS4;11 (grey bar) after 48h of incubation. Error bars represent the SD of three independent experiments. **(B)** ATPlite assay of siRNA scramble treated RS4;11 (black bar) and siRNA LINC00958 treated RS4;11 (grey bar) after 48 h of incubation. Error bars represent the SD of six independent experiments. **(C)** Cell cycle phases of siRNA scramble treated RS4;11 (black bar) and siRNA LINC00958 treated RS4;11 (grey bar) after 48 of incubation. The percentages of cells in the G0–G1, S, and G2–M phases after 48 h of active growth were reported as the mean values of three independent experiments +/− SD. **(D)** mRNA expression levels of cyclins in siRNA scramble treated RS4;11 (black bar) and siRNA LINC00958 treated RS4;11 (grey bar) after 48 h of incubation. Relative expression was determined using the 2−ΔCt method. Relative expression of cyclins is shown as mean +/− SD of three technical independent experiments. **(E)** Real Time PCR showing the TP53 expression levels in RS4;11 cells electroporated with siRNA against LINC00958 and scramble control. * = p-value < 0.05; **= p-value <0.01 Mann–Whitney t-test.
